# Sunct syndrome. Report of a case and treatment update

**DOI:** 10.4317/jced.51854

**Published:** 2015-04-01

**Authors:** Cosme Gay-Escoda, Gemma Mayor-Subirana, Octavi Camps-Font, Leonardo Berini-Aytés

**Affiliations:** 1 MD, DDS, MS, PhD. Chairman and Professor of Oral and Maxillofacial Surgery. Faculty of Dentistry – University of Barcelona. Director of the Master of Oral Surgery and Implantology (EFHRE International University/UCAM/FUCSO). Coordinating investigator of the IDIBELL institute. Head of the Department of Oral and Maxillofacial Surgery and Implantology, and Director of the TMJ Disease and Orofacial Pain Unit. Teknon Medical Center. Barcelona, Spain; 2 DDS, MS. Master degree program in Oral Surgery and Implantology. Faculty of Dentistry – University of Barcelona; 3 DDS. Fellow of the Master degree program in Oral Surgery and Implantology. Faculty of Dentistry – University of Barcelona; 4 DDS, MD, PhD. Emeritus Professor of Oral and Maxillofacial Surgery, Professor of the Master’s Degree Program in Oral Surgery and Implantology, School of Dentistry, University of Barcelona, Barcelona, Spain. Researcher of the IDIBELL Institute

## Abstract

Short-lasting unilateral neuralgiform headache attacks with conjuntival injection and tearing (SUNCT) is considered a rare trigeminal autonomic cephalgias, a group of primary headache disorders characterized by brief episodes of severe unilateral headache in the distribution territory of the trigeminal nerve, accompanied by prominent ipsilateral and cranial parasympathetic autonomic features. The present report describes a SUNCT syndrome in a 64-year-old male who had been diagnosed with trigeminal neuralgia several years ago. The patient reported stabbing pain in the orbital zone and in the left upper maxillary region, of great intensity, brief duration, and a frequency of 20-100 attacks a day. Pain episodes were accompanied by conjunctival injection and tearing. Based on the anamnesis, clinical examination and a magnetic resonance imaging scan, episodic SUNCT syndrome was diagnosed and pharmacological treatment with topiramate was started. This reduced the intensity and number of attacks to 3-6 a day.

** Key words:**Trigeminal autonomic cephalgias, SUNCT, Cluster headache, topiramate.

## Introduction

The trigeminal autonomic cephalgias (TACs) are a group of primary headache disorders characterized by brief episodes of severe unilateral headache in the distribution territory of the trigeminal nerve, accompanied by prominent ipsilateral and cranial para-sympathetic autonomic features (CPAFs) (1). TACs are comprised in section 3 of the International Classification of Headaches Disorders (1), third edition-beta (ICHD-3ß) and include: cluster headache (CH), paroxysmal hemicrania (PH), short lasting neuralgiform headache attacks, hemicrania continua (HC) and probable TAC (1). These conditions are distinguished by their attack duration and frequency, as well as response to treatment (2).

Short-lasting unilateral neuralgiform headache attacks with conjuntival injection and tearing (SUNCT) and short-lasting unilateral neuralgiform headache attacks with cranial autonomic symptoms (SUNA) are the two subsets of the ICHD-designated short lasting neuralgiform headache attacks (1). Characteristically they are moderate to severe, short-lasting strictly lateralized headaches, with prominent ipsilateral CPAFs. As for the other TACs, episodic and chronic forms of SUNCT and SUNA are reported (1). The term SUNCT is adopted when CPAFs consist exclusively of conjunctival injection and tearing (2).

SUNCT syndrome is considered a rare condition characterized by moderate or severe neuralgiform pain typically located in the area of the first division of the trigeminal nerve –though can be felt anywhere in the head. Attack episodes are very short (5 to 240 seconds) and can be described as single stabs, series of stabs or in a sawtooth pattern. As previously mentioned, such symptoms are accompanied by the CPAFs manifestations of conjunctival injection and lacrimation of the ipsilateral eye. Although attacks occur up to 60 times a day (range 3 to 200), at least 20 attacks a day with a frequency of at least one a day for more than half of the time when the disorder is active have to be reported to fulfil diagnosis criterion.  Table 1 provides the most relevant characteristics to be noted in order to establish a differential diagnosis (1-8).

**Table 1:**
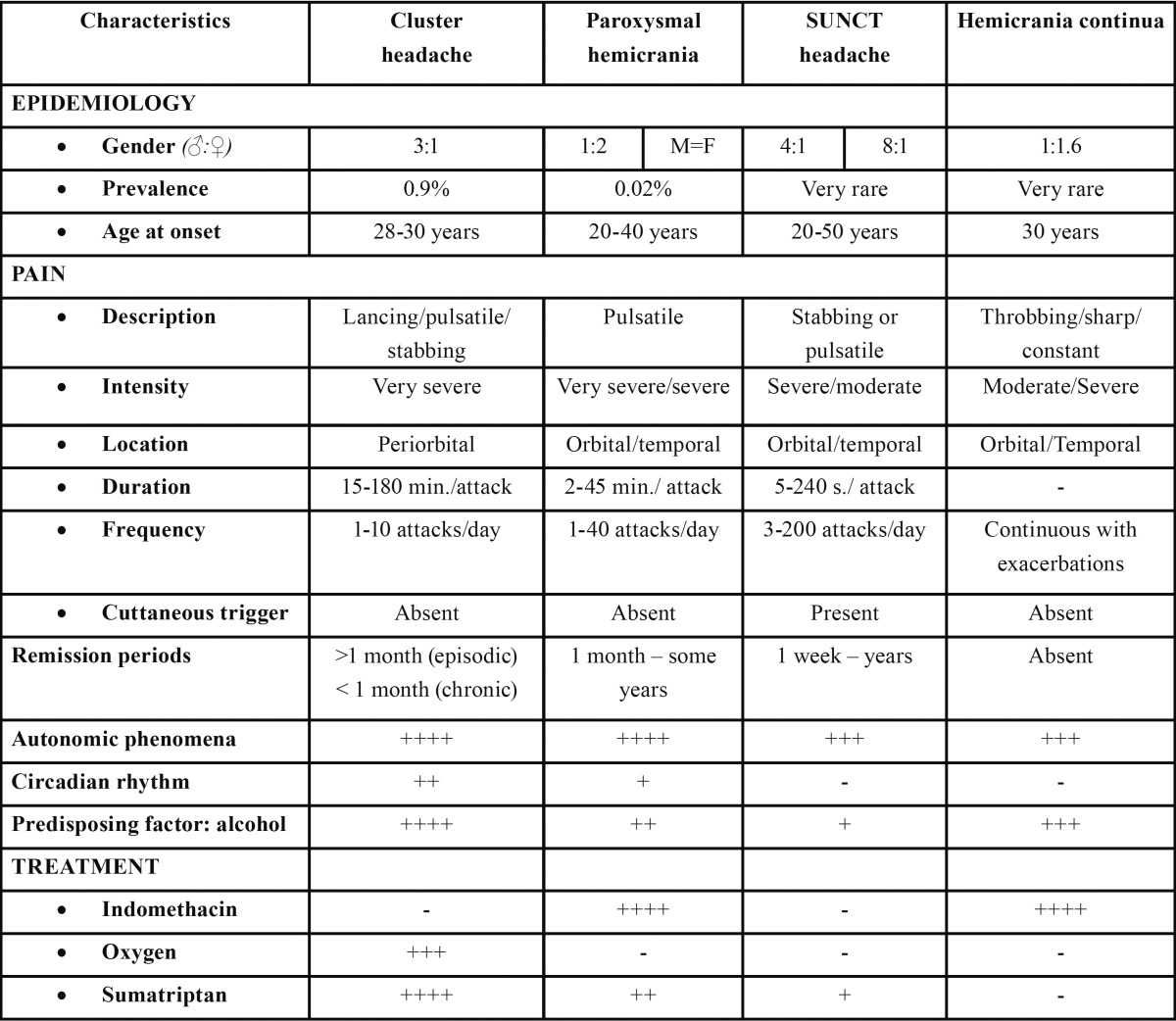
Diagnostic criteria of the trigeminal autonomic cephalgias. + rare, ++ infrequent, +++ frequent, ++++ very frequent.

The attacks may be spontaneous or can be triggered by mechanical stimuli in the territory innervated by the trigeminal nerve, without refractory periods subsequent to them in most of patients (7,8). In the course of a SUNCT attack, hyperventilation and cardiovascular changes occur before and during the attack. Additionally, increasing intraocular pressure, as well as facial and periocular temperature has been also recorded (8).

Management of this disorder is complex since no treatment is effective at all. Although intravenous lidocaine can be used as a transitional treatment until beneficial effects of a preventive treatment becomes evident, lamotrigine and topiramate are considered the most effective drugs for the preventive treatment of SUNCT (6,9,10). Response to sodium valproate, duloxetine, gabapentin, oxcarbazepine and carbamazepine has been shown less effective (6,9). In contrast to PH, indomethacin is typically ineffective, whereas oxygen and sumatriptan are unhelpful in opposition to CH (6). If drug treatment fails, surgery procedures such as ablative procedures or deep brain stimulation of the posterior hypothalamic region can be considered (9,11).

The aim of the present case report was to describe the main clinical features and treatment of a patient diagnosed with SUNCT. The study protocol was approved by the Ethical Committee for Clinical Research (CEIC) of the Dental Hospital of the University of Barcelona.

## Case Report

A 64-year-old male came to the Temporomandibular Joint and Orofacial Pain Unit of the School of Dentistry of the University of Barcelona with severe left facial pain. Patient’s pathological background comprised a stomach ulcer and arterial hypertension, controlled with omeprazole 20 mg orally (1 every 12 hours. (Omapren®, Lesvi, Sant Joan Despí, Spain)) and torasemide 10 mg orally (1 every 24 hours. (Isodiur®, Italfarmaco, Milan, Italy)), respectively. The patient had been diagnosed with trigeminal neuralgia (TN) three years ago, which was treated with carbamazepine 200 mg orally (1 ½ every 8 hours. (Tegretol®, Novartis, Barcelona, Spain)) and pregabalin 75 mg orally (1 every 8 hours. (Lyrica®, Pfizer, New York, USA)). In the last four months he had noticed an increase in the intensity and frequency of the pain that prevented him from leading a normal life. The patient presented a worsened general appearance and was depressed. Twenty days before, he had started treatment with indomethacin 25 mg orally (1 every 12 hours. (Inacid®, Merck Sharp and Dohme, New Jersey, USA)), which he stopped taking on his own initiative due to a lack of symptoms improvement.

**Figure 1:**
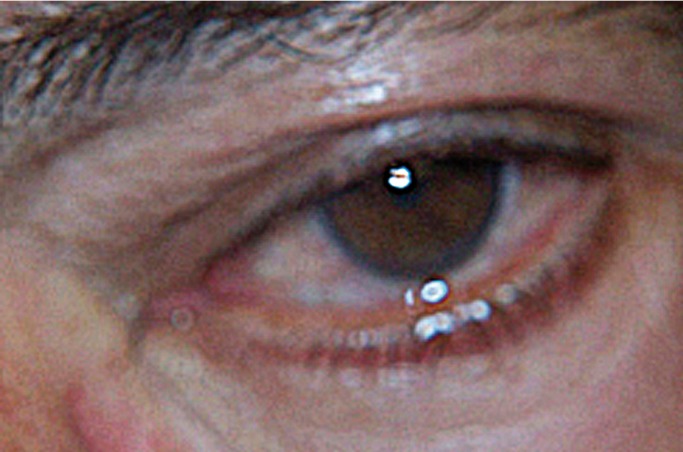
Left eye of the patient during a SUNCT syndrome attack.

Based on an exhaustive anamnesis, the pain was identified as being of an electric stabbing nature, scored as 10/10 on the visual analog scale (VAS), and located in the orbital, periorbital, frontal and upper maxillary zone of the left side of the face. The pain proved spontaneous but was also triggered by speech, chewing, swallowing, sneezing, shaving, and even simple contact with the left side of the face. The frequency of attacks during the day was 20 to 100 on average, though recently the number had increased considerably – affecting even sleep. Slight continuous discomfort was described between successive attacks. On the same visit we noted a pain attack with conjunctival injection and tearing of the left eye (Fig. 1).

**Figure 2:**
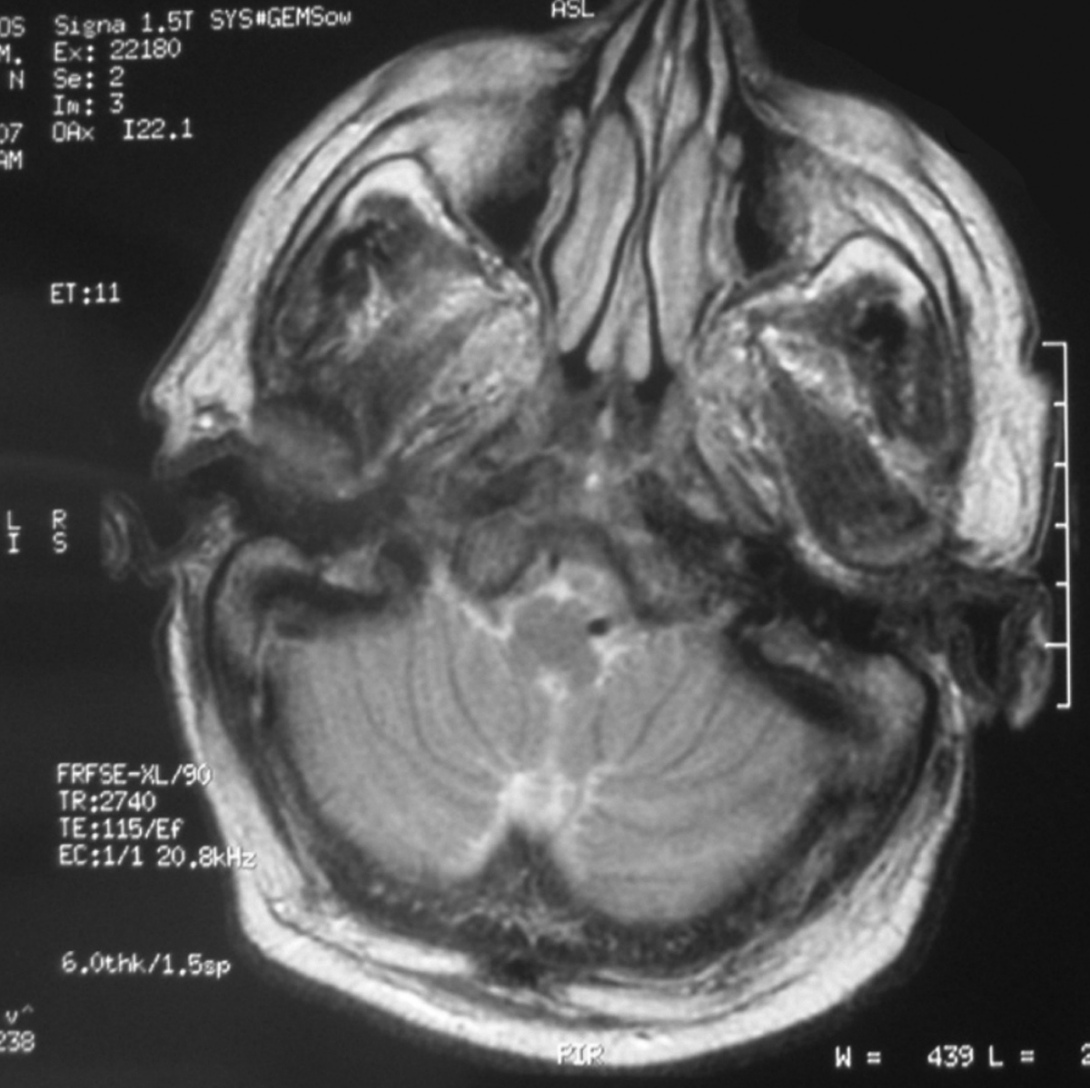
Magnetic resonance imaging scan without apparent alterations.

Following the anamnesis, a general, regional and local exploration was performed. Several complementary tests were requested: a panoramic X-ray, magnetic resonance imaging and a neurological exploration. No disease of oral or dental origin was noted, and the neurological evaluation and magnetic resonance imaging scan evaluated by a neurologist showed no anomalies (Fig. 2).

**Figure 3:**
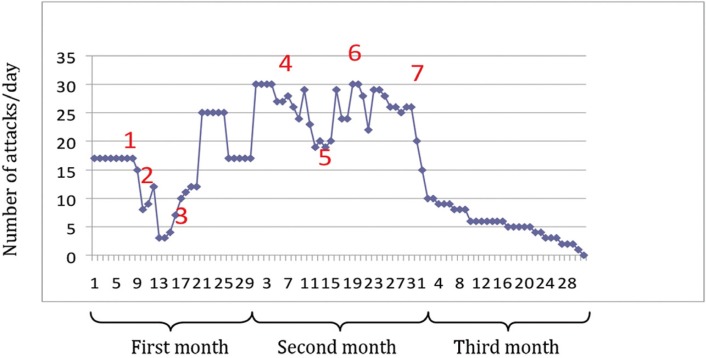
Graphic representation of the number of daily attacks recorded by the patient in the first three months of treatment with topiramate. Treatment provided: (1) Tegretol® 200 mg (0,0,0), Topamax® 25 mg (1,0,1); (2) Topamax® 25 mg (2,0,1); (3) Topamax® 25 mg (2,0,2); (4) Topamax® 25 mg (2,0,3); (5) Topamax® 25 mg (3,0,3); (6) Topamax® 25 mg (3,0,4); (7) Topamax® 25 mg (4,0,4).

Based on the data obtained, a tentative diagnosis of episodic SUNCT syndrome was established. Laboratory tests (complete blood count, renal and liver parameters and ions) requested revealed slightly elevated glutamyltransferase levels. After consultation with the personal physician of the patient, we started treatment with topiramate 25 mg orally (1 every 12 hours. (Topamax®, Janssen-Cilag, Madrid, Spain)) once informed consent was accepted. The carbamazepine dose was gradually reduced and finally suspended, while the topiramate dose was gradually increased to a dose of 200 mg a day, i.e., 4 every 12 hours. The patient was instructed to register the number of daily attacks in order to assess the effectiveness of treatment. In this way, a decrease was observed in the intensity and number of daily attacks (to 3-6 per day) (Fig. 3).

## Discussion

Since Sjaasted *et al.* (12) first described SUNCT in 1978, it has been considered a rare disorder, with an estimated prevalence of 6.6 per 100,000 and an annual incidence of 1.2 per 100,000 (10). According to the name of this syndrome (Short-lasting Unilateral Neuralgiform headache with Conjunctival injection and Tearing), SUNCT is characterized by severe, short-lasting unilateral headaches, with prominent ipsilateral conjunctival injection and tearing (1).

As in our case, SUNCT syndrome is often mistakenly diagnosed as trigeminal neuralgia (TN) because of the similarities between these entities (4,8). Both involve pain of short duration (5 to 240 seconds), in which triggering factors can be observed, such as physical contact (touch), tooth brushing or chewing, among others (2,4). The differences are that SUNCT shows a predilection for males, with clear resistance to antineuralgic drug therapy, and presents autonomic manifestations. Thermography in turn yields different patterns for TN and SUNCT. Refractory periods, which are typical of TN, have not been demonstrated in SUNCT. On the other hand, the location of SUNCT is typically periorbital – unlike TN, which in only very rare instances is confined to the territory of the first division of the trigeminal nerve. The presence or not of autonomic manifestations is of great help in establishing a correct diagnosis of SUNCT syndrome (8).

In our case the patient avoided any contact with the left upper maxilla, whether palpation of the soft tissues or dental percussion, and the possibility of tooth pain had to be ruled out initially. There have been reports of SUNCT syndrome triggered by oral stimuli such as chewing, the consumption of hot or cold beverages, and tooth brushing (5,8). A careful intraoral examination is required in order to rule out the possibility that the pain may be of dental origin –thereby avoiding unnecessary extractions or dental treatment resulting from a wrong diagnosis (13). Other potential triggering factors are facial skin stimulation (shaving, contact with cold air) and facial muscle or tongue movements. In this context, Cohen (6) reported that 79% of patients diagnosed with SUNCT presented skin stimuli as triggering factors.

Although SUNCT constitutes a primary headache, their manifestations should be in relation to another disorder such as intracranial tumours, cerebrovascular accidents injuries, arteriovenous malformations, hormonal alterations, antineoplastic therapy, human immunodeficiency virus infection or chronic active hepatitis (5,14). As in our case, detailed clinical exploration and exa-mination of the complementary tests seems to be essential in order to rule out such eventualities.

SUNCT syndrome treatment could be challenging since no treatment is effective at all and treatment recommendations are difficult to establish since controlled studies are sparse, and the disease is moreover uncommon (4). Although multiple treatment options such as gabapentin, topiramate, intravenous lidocaine and intravenous phenytoin have been proposed, lamotrigine has been considered as the drug of choice for the preventive management of SUNCT (4).

Intravenous lidocaine has been advocated for short-term prevention. This treatment offers long-lasting analgesia (up to 6 months after injection), and is of great help in acute presentations where the patient is extremely stressed and unable to lead a normal life. Intravenous lidocaine offers the advantage of obviating the need for any other medication for a certain period of time. The disadvantage is that it involves continuous intravenous infusion and thus requires cardiovascular monitorization – a situation that is scantly practical in the routine clinical setting (3,5). As regards long-term prevention of the attacks, Cohen (6) reported a 68% success rate in patients with SUNCT headache treated with lamotrigine (up to 300-400 mg/day), versus 42% with topiramate (up to 300-400 mg/day), and 45% with gabapentin (up to 3600 mg/day). Thus, the recommended first choice treatment consists of lamotrigine followed by topiramate. Other drugs such as sodium valproate and carbamazepine appear to be less effective. Carbamazepine at doses of up to 900 mg/day can offer good results when combined with prednisolone or topiramate (5).

After consultation with the personal physician of the patient, a gradually increasing administration of topiramate was performed. Although topiramate is not considered a first line treatment drug, a significantly reduction in severity and frequency of attacks was reported (Fig. 3). Topiramate is an anticonvulsant drug which exerts its action through blockade of the voltage-gated sodium channels, enhancing GABA-mediated chloride influx involving GABA-A receptor and antagonism of the glutamate kainate/AMPA receptor (15). Current dosing protocol recommends to start treatment with a low dose, followed by gradual increments until an effective dose level of 300 mg/day is reached. It is advisable to inform the patient of the need to maintain good hydration during the treatment, in order to reduce the risk of renal lithiasis and other adverse reactions related to fluid loss. During treatment with topiramate there have been reports of increased incidences of mood alterations and depression, and of a syndrome comprising acute myopia associated with secondary narrow-angle glaucoma. During treatment with this drug, patients may develop metabolic acidosis secondary to a mild to moderate reduction of serum bicarbonate concentration. This generally occurs at the start of treatment, though it also may manifest at any time in the course of therapy. For these reasons adequate patient evaluation is recommended, including the determination of serum bicarbonate (15). Our patient underwent periodic controls both by our own Service and by his personal physician, with laboratory tests every 4-6 months in order to evaluate possible side effects of the drug. Nevertheless, further research is needed to clarify the therapeutic efficacy of topiramate in SUNCT syndrome.

In cases of medically intractable primary headaches, surgical management options such as neuromodulation, involving the injection of the combination of a steroid and a local anesthetic (e.g., depomedrone 80 mg and lidocaine 2%) in the ipsilateral region of the greater occipital nerve; electrical stimulation of the occipital nerve; and deep brain stimulation in the posterior region of the hypothalamus should be considered (5,6).

Cohen (6), on the basis of functional imaging studies, recorded changes in blood oxygen load in the hypothalamus during the activation produced in the course of spontaneous SUNCT attacks. The level of activation was related to the intensity of pain. Of the 9 SUNCT patients studied, 5 presented bilateral activation while two showed activation on the side contralateral to the attacks. This resulted in the suggestion that the hypothalamus is the central generator of TACs, though this is not the case in patients with secondary SUNCT. Based on these observations, deep brain stimulation has begun to be applied to the posterior zone of the hypothalamus as a treatment strategy in severe cases of the disease, refractory to drug treatment (7,9). This technique has been applied more often in cases of cluster headache, though it is also beginning to find application in the more serious presentations of SUNCT – affording a marked reduction in pain attacks in both cases (11).

The effectiveness of hypothalamic stimulation in TACs has a number of physiopathological implications. The posterior zone of the hypothalamus receives information from the trigeminal territories through the direct trigeminal-hypothalamic pathway. Direct electrical and peptidic stimulation of the posterior area of the hypothalamus modulates the activity of the caudad nucleus of the trigeminal nerve. This explains why hypothalamic stimulation can restore hypothalamic modulation of the trigeminal caudad nucleus, preventing activation of the trigeminal reflex that is believed to be responsible for the pain and autonomic symptoms. This in turn supports the hypothesis that hypothalamic stimulation modulates the activity of the caudad nucleus of the trigeminal nerve, which controls the above mentioned trigeminofacial reflex of SUNCT (6,7,10,11). This seems to open the way to future treatment options, though it must be accepted that hypothalamic activity may not be the only mechanism involved in SUNCT. In order to reduce risks, electrical stimulation of the occipital nerve has been proposed – with good results in patients with cluster headache and paroxysmal hemicrania (6). Thus, the future of the treatment of this type of headache is based upon the investigation of its underlying physiopathological mechanisms.
